# Machine learning and molecular descriptors enable rational solvent selection in asymmetric catalysis†
†Electronic supplementary information (ESI) available: Library of solvents and molecular descriptors. See DOI: 10.1039/c9sc01844a


**DOI:** 10.1039/c9sc01844a

**Published:** 2019-05-30

**Authors:** Yehia Amar, Artur M. Schweidtmann, Paul Deutsch, Liwei Cao, Alexei Lapkin

**Affiliations:** a Department of Chemical Engineering and Biotechnology , University of Cambridge , Philippa Fawcett Drive , Cambridge , CB3 0AS , UK . Email: aal35@cam.ac.uk; b Aachener Verfahrenstechnik – Process Systems Engineering , RWTH Aachen University , Aachen , Germany; c UCB Pharma S.A. Allée de la Recherche , 60 1070 , Brussels , Belgium; d Cambridge Centre for Advanced Research and Education in Singapore Ltd. , 1 Create Way, CREATE Tower #05-05 , 138602 , Singapore

## Abstract

Rational solvent selection remains a significant challenge in process development. Here we describe a hybrid mechanistic-machine learning approach, geared towards automated process development workflow. A library of 459 solvents was used, for which 12 conventional molecular descriptors, two reaction-specific descriptors, and additional descriptors based on screening charge density, were calculated. Gaussian process surrogate models were trained on experimental data from a Rh(CO)_2_(acac)/Josiphos catalysed asymmetric hydrogenation of a chiral α-β unsaturated γ-lactam. With two simultaneous objectives – high conversion and high diastereomeric excess – the multi-objective algorithm, trained on the initial dataset of 25 solvents, has identified solvents leading to better reaction outcomes. In addition to being a powerful design of experiments (DoE) methodology, the resulting Gaussian process surrogate model for conversion is, in statistical terms, predictive, with a cross-validation correlation coefficient of 0.84. After identifying promising solvents, the composition of solvent mixtures and optimal reaction temperature were found using a black-box Bayesian optimisation. We then demonstrated the application of a new genetic programming approach to select an appropriate machine learning model for a specific physical system, which should allow the transition of the overall process development workflow into the future robotic laboratories.

## Abstract 2

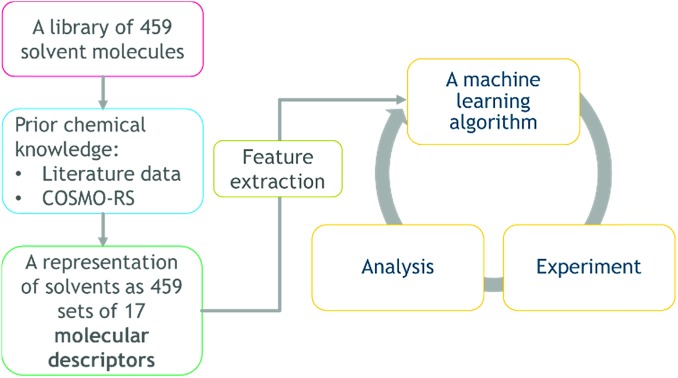
Rational solvent selection remains a significant challenge in process development.

## Introduction

Process development of new reactions is a challenging, complex, and expensive task.^1^ Traditionally, process chemists rely on intuition and past experience to navigate reaction conditions and select solvents, although various design of experiments (DoE) methodologies are increasingly being adopted, mainly in industrial process development labs.^2^ Bayesian optimisation-based DoE has recently emerged as a new method of process optimisation.^3^–^9^ The statistical algorithms used are able to rapidly learn the complex reaction behaviour and optimise the desired outcomes by modulating process conditions such as temperature, reaction time, and feed concentrations. This approach works well in the case of continuous variables, but not so well in the case of discrete variables, such as choice of catalyst, substrate, or solvent, that play a crucial role in most reaction optimisation studies.^10^ Only very recently have discrete variables been considered in self-optimisation frameworks, but without physical representation.^11^ The difficulty in representing discrete variables, as well as the ‘curse of dimensionality’, makes it challenging to treat them algorithmically without resorting to expensive high-throughput experimentation.^12^–^14^ A potentially useful way to resolve this problem is to use molecular descriptors^15^,^16^ to introduce physically meaningful continuous variables linking discrete variables. In this study we find that solvent descriptors can indeed be incorporated into the reaction self-optimisation paradigm to create predictive surrogate models, thereby dramatically enhancing process development workflows for solvent selection in practice, as well provide new generic mechanistic insights about the specific reaction of interest.

Attempts to utilise generalisations of fundamental physical knowledge of discrete variables can be traced back to the 1950s when Taft demonstrated that steric effects can be isolated, and developed some of the first steric parameters.^17^ This paved the way for ligand and substrate descriptors,^18^,^19^ which are now common in multivariate linear regression models, and mechanistic interrogation.^20^–^23^ Successful development towards solvent optimisation has been far more limited. Reizman and Jensen have attempted to bypass physical knowledge of solvents in their microfluidic reaction droplets platform.^24^ A flow experiment was combined with an algorithm based on sequential adaptive response surface methodology and optimal DoE. The algorithm identified DMSO as a promising solvent out of a pre-selected set of 10 solvents, for the alkylation of 1,2-diaminocyclohexane. As a black box method, it does not provide physical insights.

Exploring the solvent landscape more extensively, and simultaneously gaining physical insights, will almost certainly require a combination of statistics and chemical information. Murray *et al.* make use of a linear dimensionality reduction approach,^25^ first demonstrated for solvents in the 1980s separately by Chastrette *et al.*^26^ and Carlson *et al.*,^27^ to parametrise a solvent map using molecular descriptors. Murray *et al.* used this concept to obtain a solvent map and thus extend the traditional factorial DoE approaches. In this study we adopted the Principal Component Analysis (PCA) approach as one option for extracting features,^28^ or meaningful input variables, from the large dimensional descriptor space for the machine learning surrogate models. This is also referred to as feature engineering.^29^,^30^ In addition to the previous study on molecular descriptors of solvents,^25^ we also compute more reaction-specific descriptors, in order to produce more relevant features.

Struebing *et al.* describe a computer-aided molecular design strategy aimed at utilising physical knowledge that is conceptually similar to the one presented here.^31^ They used quantum mechanics to compute reaction rate constants in six initial solvents and constructed a qualitative surrogate model based on a linear free-energy relationship between the rate constant and five molecular descriptors. The subsequent step used mixed-integer linear programming to select the next solvent, whose performance was predicted *via* quantum mechanics as well. This single-objective method was tested on the Menschutkin reaction. Ultimately, nitromethane was identified and verified as the superior solvent. The rate constants for the solvents were shown to exhibit an approximate proportional relationship with the dielectric constant. This demonstrates the potential of a hybrid mechanistic-machine learning approach: it incorporates general prior knowledge into the data analysis framework, rather than leaving it to the algorithm to ‘rediscover’ this information.

**Fig. 1:**
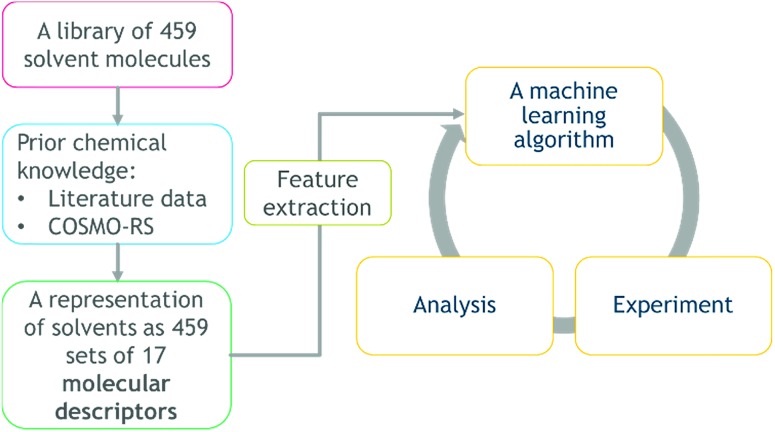
The workflow and transition of chemistry knowledge into machine learning domain and process domain, *i.e.*, experiment and analysis.

The method developed in this study extends the growing use of machine learning in chemistry and chemical engineering,^9^,^32^ specifically an attempt to combine multi-objective DoE algorithms with physical knowledge in the form of solvent properties. The workflow and transition of chemistry knowledge into machine learning domain and process domain is illustrated in Fig. 1. We start with a library of 459 candidate solvents. Then, we acquire physical knowledge from property databases and through molecular simulations leading to 17 molecular descriptors. The transition of physical knowledge into the machine learning domain is achieved *via* a dimensionality reduction that provides features for the Gaussian process machine-learning models then used in Bayesian optimisation with lab experiments and analysis in the loop. The obtained results could be linked back to the physically-meaningful molecular descriptors and represent new, generic physical knowledge. The strategy is computationally inexpensive, in that it does not require high performance computing facilities. Furthermore, it is applicable to multi-objective problems, and to difficult reactions, exemplified here with a transition metal catalysed asymmetric hydrogenation.

**Scheme 1:**
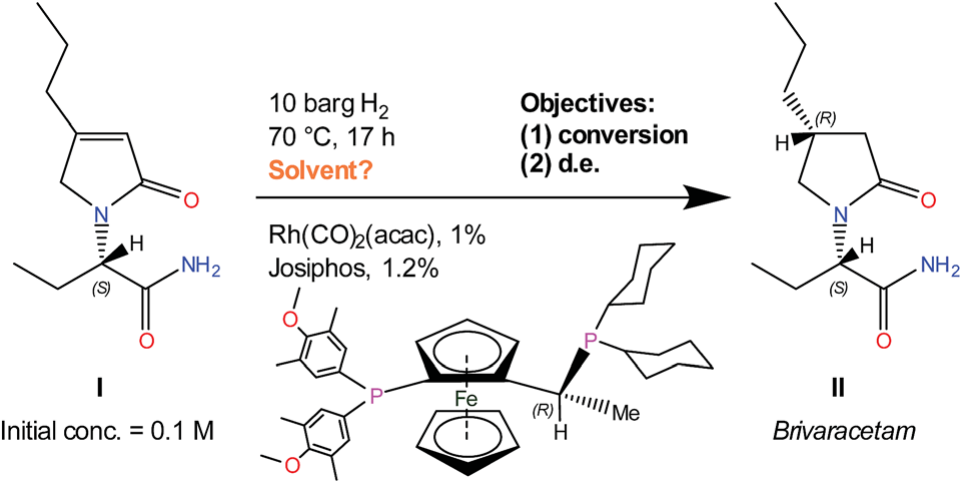
Rh–Josiphos catalysed asymmetric hydrogenation reaction of **I**, conditions, and objectives.^34^,^35^

The example reaction under study is a Rh(CO)_2_(acac)/Josiphos(cyclohexyl/4-methoxy-3,5-dimethylphenyl) catalysed asymmetric hydrogenation of a complex chiral α-β unsaturated γ-lactam (**I**), shown in Scheme 1, used to produce UCB Pharma's new anti-epileptic drug Brivaracetam (**II**).^33^

The aim is to develop a workflow, which reduces experimental bias and, hence, explores non-intuitive solvent selections, leads to a predictive surrogate model which can be used in an optimisation, and also helps to develop new generic physical knowledge.

**Fig. 2:**
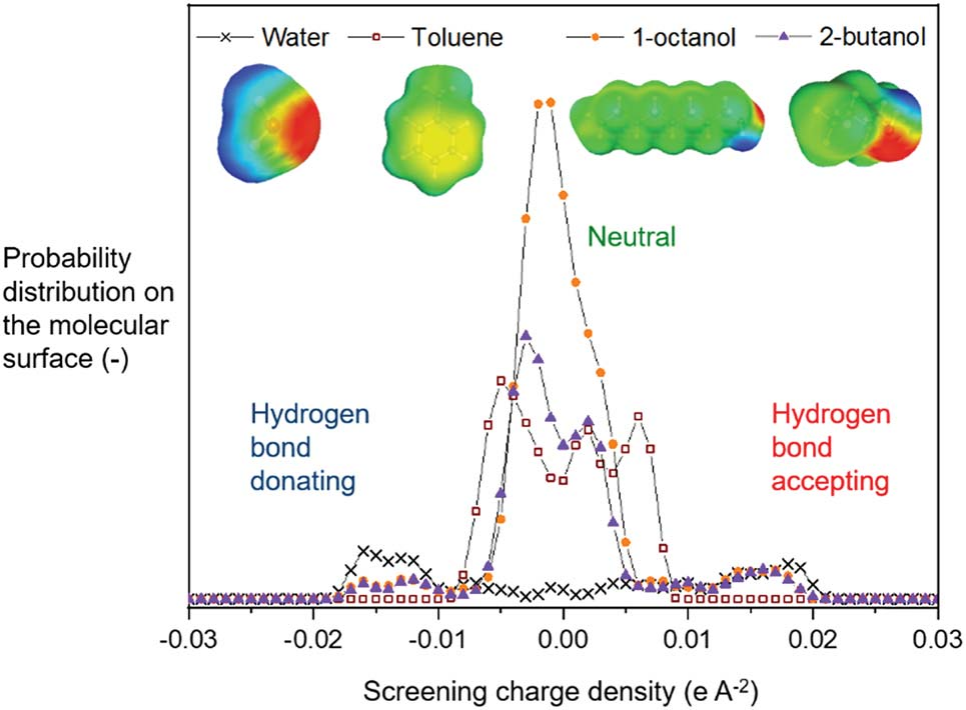
Screening charge density profiles (‘*σ* profiles’) of four example solvents.

Physical knowledge about solvents is introduced to the machine learning context *via* a set of molecular descriptors. Apart from descriptor databases available in the literature, a source of descriptors can be computational software, such as COSMO*therm*, which combines quantum chemistry and thermodynamics to predict properties. COSMO*therm* has been used since the 1990s for a range of applications, such as the evaluation of new solvents for solid–liquid extraction of biopharmaceuticals,^36^ partitioning of organic substances into different phases,^37^ and predicting solute partition in multiphase complex fluids.^38^ Among the descriptors computed in this study, we explored screening charge density profiles.^39^,^40^ These information-rich ‘*σ* profiles’, which are histograms of screening charge density on the molecular surface, were converted to numerical descriptors per solvent, each defining a different segment of the profile, see Fig. 2. This concept has previously been used in multilinear regression models to correlate CO_2_ absorption and desorption capacities in various amine solvents,^41^ as well as rate constants in the case of a Diels–Alder reaction.^42^ This work was later extended to identify solvents that possess the best reaction performance under model uncertainty,^43^ which allows to explicitly account for uncertainty in the process of descriptor design, and further solvent selection/design workflow.

This paper comprises three sections. First, we present a hybrid mechanistic-machine learning approach to identify promising solvents in a large decision space. For this we evaluate different descriptors: (i) *σ* profiles only, and (ii) a dimensionality-reduced set of a larger set of 17 meaningful molecular descriptors. Then, we focus on the promising solvents identified in the first part, and algorithmically determine operating conditions (temperature and solvent mixtures). Thus, this work presents a two-step approach, utilising advantages of both *a priori* knowledge, and black box optimisation. Finally, we incorporate into the workflow of solvent selection an automated tool for determining optimal machine learning pipelines.

## Materials and methods

### Experimental

Experiments were conducted in an argon-filled Vigor glovebox in a stainless-steel screening autoclave (Cat7, HEL), using 10 mL glass reaction vials (as illustrated in ESI†). The substrate, catalyst precursor and ligand were weighed and charged into the reactor, followed by the solvent and a magnetic stirrer. The autoclave was sealed and purged with H_2_ three times, before being pressurised slowly to 10 barg and heated to 70 °C. Stirring rate was 1000 rpm, and material loadings were 0.1 M [**I**]_0_, 1% Rh(CO)_2_(acac), and 1.2% Josiphos. Reaction time was 17 h for all experiments. Conversion and diastereomeric excess (d.e.) were determined by chiral HPLC (Shimadzu Prominence, Chromspher column by Agilent, 8 min run time, 1 mL min^–1^ flow rate, 22 °C column temperature, acetonitrile : H_2_SO_4_ (98 : 2 v/v%) mobile phase). As the quality of machine learning models depends heavily on the quality of the training data, experiments were repeated two or three times; experimental error was determined and is shown with the results. Initially we screened 34 solvents from a diverse range of solvent classes, guided by expert knowledge. The aim was to cover as many types of solvents, but using molecules selected by expert synthetic chemists, based on their prior experience. From these we used the descriptors of 25 solvents as inputs to the algorithm in order to test the predictions on the other 9. There are numerous methods of selecting initial data sets; this expert-guided strategy allows for a comparison of algorithm-inspired suggestions and human expertise.^44^ The objectives of interest were conversion and d.e. We note that using ΔΔ*G*^‡^ (difference in Gibbs free energy between diastereomeric transition states, commonly employed in data analysis for asymmetric catalysis), showed little difference in the models, compared to d.e. For this case study, we determined *a priori* that the relation between solvents is approximately consistent across most continuous variables, such as temperature, so that an initial focus on the discrete variable only, with other conditions held constant, is appropriate.

### Molecular descriptors

From the available solvents library^43^ we removed entries with no data, those that were very similar and also solvents with boiling points at 10 bar(g) significantly below the reaction temperature, as this would result in a high-pressure process. This resulted in a library of 459 solvents. For each solvent in the library we created a set of 17 molecular descriptors. These, as well as their sources, are listed in Table 1. Descriptors were either taken from literature,^45^ or calculated in COSMO*therm*.^39^ Properties were computed at 25 °C, except for the reaction-specific properties (Henry's constant of H_2_, and ln(*γ*) of **I**), which were computed at 70 °C. For calculating screening charge density profiles (‘*σ* profiles’), structures were taken from the COSMO*therm* 1401 database with BP-TZVP-COSMO parametrisation. We converted *σ* profiles to numerical descriptors by segmenting them into *n* segments, and calculating the area under the curve for each segment. We evaluated models based on *n* = 3 (segmented at *σ* = –0.010 and 0.010 e A^–2^, as illustrated in Fig. 2), and *n* = 5 (segmented at *σ* = –0.015, –0.005, 0.005, and 0.015 e A^–2^). In models 3–6 PCA was used to reduce the dimensionality of 17 descriptors to generate the first four principal components *t*_1_–*t*_4_, capturing 70% of the variance in the original descriptors. In these models 
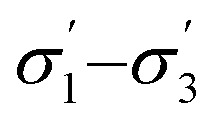
 was used in preference over *σ*_1_–*σ*_5_ within the 17 descriptors.

**Table 1:** List of solvent molecular descriptors used in this work

Descriptor (units)	Source
Molecular weight (g mol^–1^)	Stenutz^45^
Density (g mL^–1^)	Stenutz^45^
Molar volume (mL mol^–1^)	Stenutz^45^
Refractive index (—)	Stenutz^45^
Molecular refractive power (mL mol^–1^)	Stenutz^45^
Dielectric constant (—)	Stenutz^45^
Dipole moment (*D*)	Stenutz^45^
Melting point (°C)	Stenutz^45^
Boiling point (°C)	Stenutz^45^
Viscosity (cP)	COSMO*therm*^39^
ln *P*_octanol–water_ partition coefficient (—)	COSMO*therm*^39^
Vapour pressure (mbar)	COSMO*therm*^39^
Henry's constant of H_2_ in solvent (bar)	COSMO*therm*^39^
ln(*γ*) activity coefficient of **I** in solvent (—)	COSMO*therm*^39^
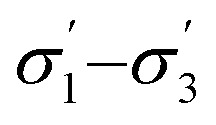 profiles segmented into three (—)	COSMO*therm*^39^
*σ*_1_–*σ*_5_ profiles segmented into five (—)	COSMO*therm*^39^
*t*_1_–*t*_4_: principal components from PCA (—)	—

### Machine learning algorithm

The recently published Thompson sampling efficient multi objective optimisation (TS-EMO) algorithm, described in detail elsewhere,^46^ was used. The algorithm's utility in chemical process optimisation was recently demonstrated in an *in silico* multi-objective optimisation case study,^47^ and for purely experimental self-optimisation in synthetic chemistry.^9^ A major advantage of this algorithm is its ability to treat multiple objectives independently, as the trade-offs in process objectives are often complex, and are usually addressed through scalarisation methods that have their own drawbacks.^48^ When trained on initial experimental data, TS-EMO builds a Gaussian process (GP) surrogate model for each objective. GPs have been shown in the literature to be able to adequately represent chemical and bio-processes.^9^,^49^ Then, TS-EMO samples from this model using Thompson sampling,^50^ to approximate the Pareto set of optimal solutions, and subsequently identifies the points which maximise the hypervolume of the statistical surrogate model. Thompson sampling enables the suggestions to meet the ‘exploration and exploitation’ goal: to optimise, as well as to suggest points that reduce the model uncertainty in the unexplored regions of the parameter space. By running the simulations multiple times and identifying the most often-suggested solvents, one can bias this paradigm more towards exploitation. This closed-loop optimisation procedure is conducted until new and superior solvents are identified. The original TS-EMO algorithm is designed to work with continuous variables; our modifications include a removal of the genetic algorithm within TS-EMO (which identifies the next sampling point). This was replaced by an exhaustive enumeration of all possible solvents (making it faster since we only evaluated GP samples of the 459 candidate points). Unlike high-throughput screening,^51^,^52^ or expert-guided approaches, this machine learning method strategically and resourcefully guides experimentation, and utilises each data point in the determination of the next best experiment.

An overview of the six models considered in this study is given in Table 2. Models 1–3 are used for DoE, and then models 4–6 are compared to model 3 to investigate model robustness when less chemical information is used.

**Table 2:** Comparison of different GP surrogate models for conversion using 58 solvent data (leave-one-out cross validated). *t*_*i*=1–4_ = principal components, reduced from 17 descriptors. *q*^2^ refers to the cross-validated correlation coefficient

Model	Descriptors	*q*_conversion_^2^
Model 1	*σ*_1_, *σ*_2_, *σ*_3_, *σ*_4_, *σ*_5_	0.61
Model 2	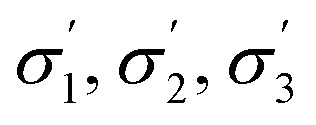	0.76
Model 3	*t*_1_, *t*_2_, *t*_3_, *t*_4_	0.81
Model 4	*t*_1_, *t*_2_, *t*_3_	0.84
Model 5	*t*_1_, *t*_2_	0.77
Model 6	*t*_1_	0.63

In the solvents mixing study, the optimisation variables were (i) temperature, and (ii) solvent volume fractions *x*_*i*=1,2,3_. In total, four solvents were mixed, but only three were used as variables to avoid over-specifying the problem. The optimisation was conducted as a batch-sequential optimisation (five reactions per batch). To do this, two further algorithm modifications were made. First, we note that the reactor used does not allow different temperatures for parallel reactions in the same batch. Therefore, because temperature is one of the optimisation variables, we ran the algorithm in two steps. Step 1 is the normal algorithm run, using all variables including the reaction temperature, generating one recipe (‘recipe’ being the set of suggested reaction conditions to be tested experimentally). Step 2 is the application of the normal batch-sequential method in TS-EMO but holding temperature at the previous selection, and, hence, selecting four more recipes at that temperature. In Step 2 we also included the constraint that the solvent fractions *x*_*i*=1,2,3_ must sum to less than 1, so that the fraction of the fourth solvent would be the balance. As this did not work with the NSGA-II implementation normally used in TS-EMO, we switched to the ‘gamultiobj’ from the ‘Global Optimisation Toolbox’ within Matlab. Finally, we used the classification methodology Tree-based Pipeline Optimisation Tool (TPOT), which is described elsewhere;^53^ its source code is available on GitHub.

## Results and discussion

### *σ* profiles as solvent molecular descriptors (models 1 and 2)

**Fig. 3:**
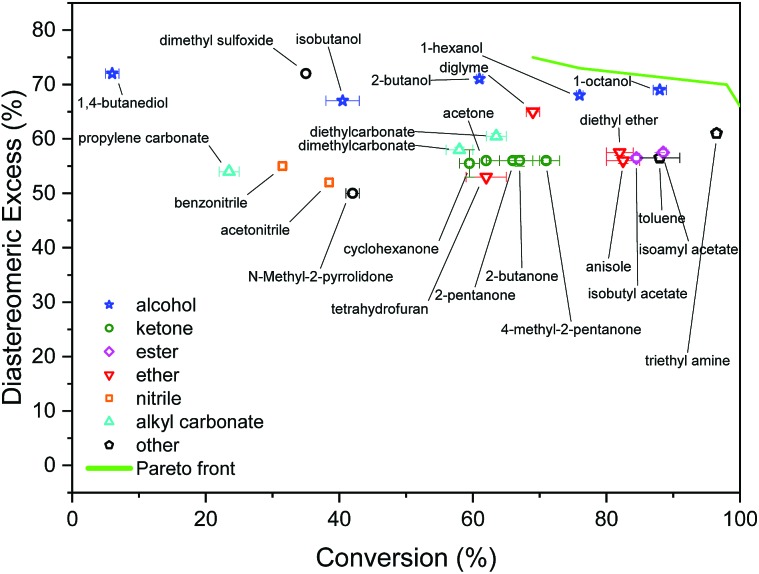
Results of initial screening of solvents from diverse classes.

The initial screening comprised solvents from diverse classes, selected by experienced synthetic chemists based on prior knowledge, see Fig. 3. The results show a wide range of outcomes for conversion, and a moderate range for d.e. Only one solvent from this set resulted in conversion above 90%, and no solvent lies on the approximation of the Pareto front, which is the set of non-dominated points (those that cannot be improved in one objective without a deterioration in the other objective), determined later in the study. When trained on this data using model 1 input descriptors (*σ*_1_–*σ*_5_), the next solvents suggested by the algorithm were dibutyl amine, methyl octanoate, eucalyptol, and ethyl acetate. Experimentally, the first three of these solvents resulted in reactions with conversions above 90%. The information contained in the outcomes of experiments with these solvents significantly improves the surrogate model, as shown on a test set before and after the inclusion of the new solvents (see Fig. S1 and S2†). These results were incorporated into the algorithm, and the algorithm was re-trained, which then identified aniline, methyl pentanoate, propyl propanoate, and butyronitrile, to be tested experimentally in the next iteration. Table S1† shows detailed results of reaction outcomes with all new solvents.

The hyperparameters of the surrogate model trained on all solvents tested in this study, shown in Table 3 (model 1, entries 1–5), give information on the impact of each input variable on each objective. This is known as automatic relevance determination, and refers to the length scales that parametrise the covariance matrix.^54^ The lower the value, the greater is the significance of the variable. Thus, *σ*_3_ (model 1, entry 3) bears the greatest influence on conversion, while *σ*_1_, *σ*_2_, and *σ*_5_ are most decisive towards d.e. One can rationalise this by noticing that the alcohols in Fig. 3 consistently show approximately 70% d.e., as they contain the same hydrogen bond accepting descriptor in the *σ* profiles, which is the information contained in *σ*_5_. Solvents of a similar class, such as alcohols and ketones, tend to cause similar d.e., information mostly contained in the extremes of a *σ* profile (*σ*_1_ and *σ*_5_). On the other hand, the neutral segment *σ*_3_ impacts mainly conversion, which is demonstrated by the differences in conversion reached with butanol, hexanol, and octanol.

**Table 3:** Surrogate model hyperparameters using different models. GP1 is for conversion, GP2 is for d.e. Values refer to the length scales that parametrise the covariance matrix (automatic relevance determination). Most impactful variables are shown in bold. Note that hyperparameters are to be compared between variables within each model, not between different models

Model	Variable	GP1	GP2
Model 1: *σ*_1_–*σ*_5_	*σ*_1_	4.41	**0.44**
Model 1: *σ*_1_–*σ*_5_	*σ*_2_	6.86	**0.41**
Model 1: *σ*_1_–*σ*_5_	*σ*_3_	**1.07**	1.66
Model 1: *σ*_1_–*σ*_5_	*σ*_4_	10.21	3.75
Model 1: *σ*_1_–*σ*_5_	*σ*_5_	2.92	**0.82**
Model 2: 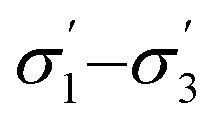	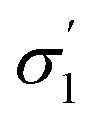	2.05	**0.15**
Model 2: 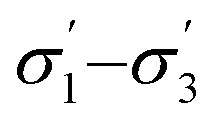	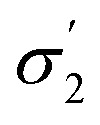	**0.89**	2.21
Model 2: 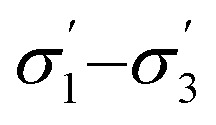	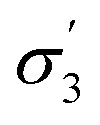	2.17	**0.03**
Model 3: *t*_1_–*t*_4_	*t*_1_ ( 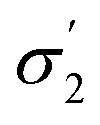 , *R*, ln *P*, *v*_M_)	**1.23**	**0.33**
Model 3: *t*_1_–*t*_4_	*t*_2_ (*T*_B_, *T*_M_)	2.53	1.07
Model 3: *t*_1_–*t*_4_	*t*_3_ (*ρ*, 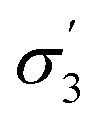 )	2.42	0.74
Model 3: *t*_1_–*t*_4_	*t*_4_ (ln(*γ*))	5.51	**0.11**
Model 4: *t*_1_–*t*_3_	*t*_1_	**1.33**	0.12
Model 4: *t*_1_–*t*_3_	*t*_2_	2.80	0.08
Model 4: *t*_1_–*t*_3_	*t*_3_	2.19	**0.06**
Model 5: *t*_1_–*t*_2_	*t*_1_	**1.73**	0.95
Model 5: *t*_1_–*t*_2_	*t*_2_	3.28	0.76
Model 6: *t*_1_	*t*_1_	**1.14**	**0.89**
Mixing model	*x*_1_	**1.97**	4.76
Mixing model	*x*_2_	15.35	31.62
Mixing model	*x*_3_	31.62	8.52
Mixing model	*T*	**0.30**	**2.52**

When *σ* profiles were segmented into only three regions instead of five (model 2), the initial model trained on the same training data is significantly more accurate for conversion, as shown in Fig. S3.† The advantage of a smaller number of input variables in the surrogate model outweighs the potential loss of fidelity of chemical information through wider ranges of screening charge densities used. Indeed, using this model, some of the same solvents are suggested, including methyl pentanoate and propyl propanoate, and some new ones such as 5-nonanone and 1-nonanol (Table S2† shows detailed results of the outcomes). Both new solvents gave ≥90% conversion, and 1-nonanol is amongst the best solvents found in this reaction, with 70% d.e. A further iteration led to experimentally testing *tert*-butylamine.

Unlike conversion, d.e. is challenging to predict accurately, due to the limited range of the data. However, d.e. predictions appear to be better using model 1 (*σ*_1_–*σ*_5_) than model 2 (
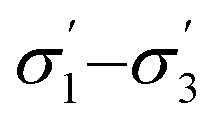
). We have already identified that solvents primarily affect reactivity and only in part, d.e. Therefore, a model based on solvent descriptors cannot be expected to be accurate for d.e. in this case. Thus, it is more useful to think of d.e. prediction as a classification problem (50–60% as ‘low’, 60–70% as ‘high’). Using model 2, the final model correctly predicts the class of only five out of the nine data points, whereas seven out of nine are correctly classified using model 1. Solvent polarity, which is information contained in the profile extremes (non-neutral regions), is more impactful towards d.e. and is better described by *σ*_1_–*σ*_5_ than 
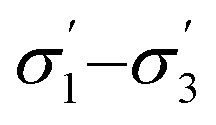
. Hyperparameters using 
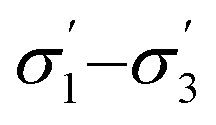
 are shown in Table 3 (model 2, entries 6–8).

### Solvent molecular descriptors based on PCA (models 3–6)

Selecting to use only-profiles as descriptors we introduce a descriptor-bias. To overcome this we use a large set of descriptors and identify a set of the reduced dimensionality composite descriptors that can practically be used in ML-optimisation workflow. Utilising the set of all literature and calculated descriptors, shown in Table 1, PCA was used to reduce the dimensionality, and the first four principal components were used as inputs in TS-EMO (model 4). The base descriptors shown in parenthesis for model 3 in Table 3 indicate what properties are approximately described by each principal component, as determined by a correlation analysis. The algorithm selects for 2,6-dimethyl-4-heptanone, which was tested experimentally, and gives >90% conversion. Additional solvents leading to excellent conversions selected by this method include 2,4-dimethyl pentane, propyl benzene, mesitylene, cumene, and tributyl amine, see Table S3.† Evidently the method learns of promising solvents and suggests them to the human researcher, providing a workflow that may elude expert intuition. Many algorithm-inspired solvents have outperformed the outcomes achieved using the initial human-selected solvents, as shown in Table 4. We note that results of a human strategy are based on the level of expertise in this particular case and are not universal across all researchers.

**Table 4:** Comparison of algorithm performance *vs.* human intuition: number of solvents found in each strategy showing outcome that is high in conversion (>90%), high in d.e. (>60%), and high in both objectives

Strategy	Conversion > 90%	d.e. > 60%	Conversion > 90% and d.e. > 60%
Human	1 of 34 (3%)	13 of 34 (38%)	1 of 34 (3%)
Algorithm	12 of 18 (67%)	10 of 18 (56%)	7 of 18 (39%)

The surrogate model for conversion that uses PCA (model 3) is superior to the model that uses *σ* profiles only (models 1 and 2). This is likely because model 3 includes further physico-chemical information, such as viscosity and solubility of a reactant. These descriptors capture information regarding phenomena that affect reaction conversion, such as mass transfer and molecular interactions. This highlights the importance of developing correct molecular descriptors for a specific reaction of interest. Model 3 does not improve the d.e. prediction; the additional descriptors do not contain extra important information for d.e., as compared to models 1 and 2. The hyperparameters are shown in Table 3 (model 3, entries 9–12), as well as the descriptors that most contribute to the principal components.

Whilst excellent conversions (>90%) could be achieved, attaining high d.e. proved to be more challenging, as the behaviour rendering this outcome requires specific interactions between the chiral ligand, and the substrate, and d.e. in this case is unlikely to reach >75% in any solvent under the same conditions for temperature and pressure. Therefore, the choice of chiral ligand is also key in this case, rather than just the solvent. The highly promising solvents described above are rarely reported in the vast body of literature on asymmetric hydrogenation. This is partially due to experimenter bias, and due to ease of access to the most commonly used laboratory reagents. Certainly, further analysis of the suggested solvents is required, specifically with respect of cost, supply chain and downstream separation.

### Evaluation and cross-validation of different surrogate models

**Fig. 4:**
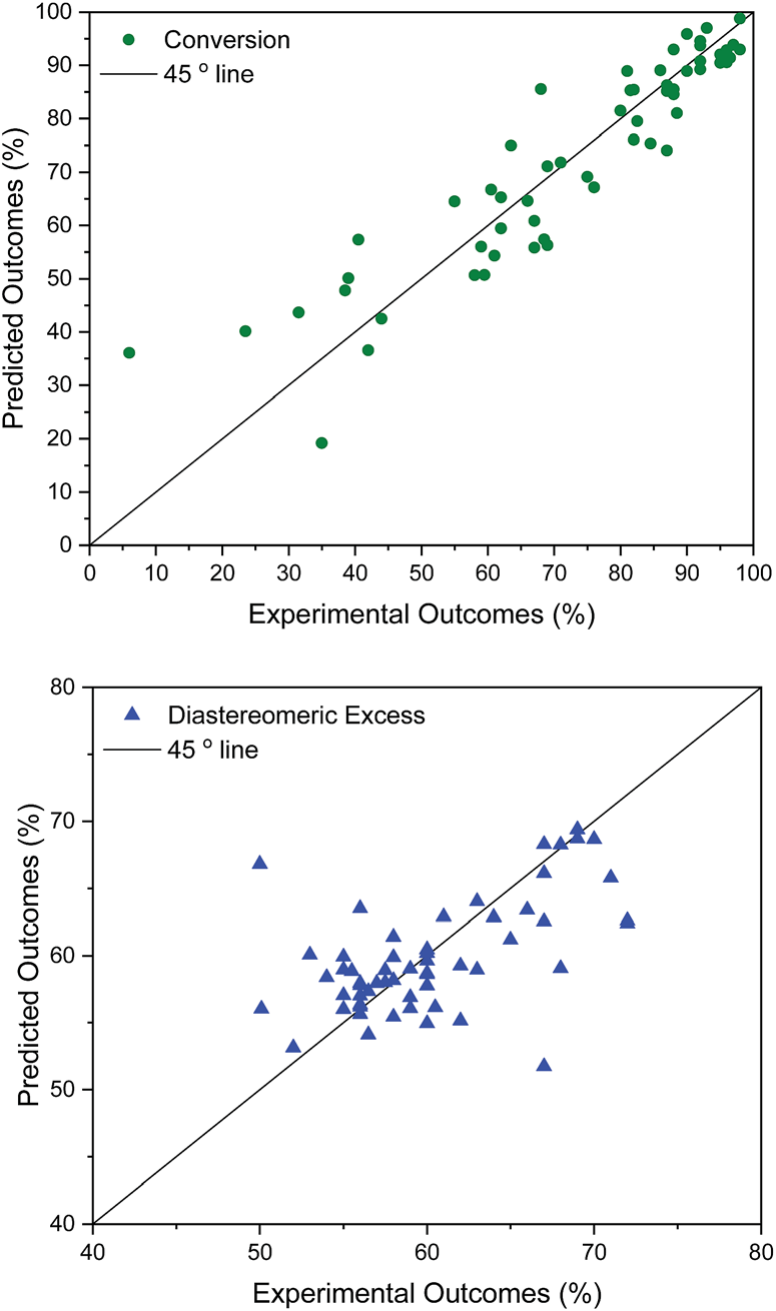
Leave-one-out cross validated surrogate model for conversion, *q*^2^ = 0.84 (top: using model 4 input descriptors) and d.e. (bottom: using model 1 as input descriptors), *q*^2^ = 0.33 (73% correctly classified, as >60% or <60%).

The developed surrogate models described above are statistically predictive. It was not entirely expected that the model based on continuous GPs with the chosen molecular descriptors would correctly model discrete variables in such a complex reaction. Nonetheless it works reasonably well: model robustness and predictive ability was investigated using leave-one-out cross-validation,^55^ as summarised in Table 2. For this, all 58 data points, selected by a human experimentalist and by the algorithm, were included. The model robustness for conversion is best in model 4 using *t*_1_–*t*_3_ (*q*^2^ = 0.84), followed by model 3 (*q*^2^ = 0.81), capturing only 62% of the variance in the original data, model 5 (*q*^2^ = 0.79), model 2 using 
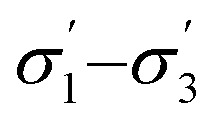
 (*q*^2^ = 0.76), model 1 using *σ*_1_–*σ*_5_ (*q*^2^ = 0.61), and finally using just one parameter, *t*_1_ (model 6, *q*^2^ = 0.59), capturing only 32% of the variance in the original data. For d.e., model 1 is best (*q*^2^ = 0.33), predicting 73% of the solvents to be in the correct category (‘high’ *vs.* ‘low’ d.e.), whereas the other models are weak. The cross-validated predictions for the best models for conversion and d.e. are shown in Fig. 4. These results suggest that an ensemble of GP models, each based on different descriptors, provides better results than using the same descriptors for the different objectives.

### A solvent mixing black box approach

The concepts discussed so far demonstrate algorithmic navigation of the solvent space using molecular descriptors. As a next step, we zoned in on the promising solvents and investigated algorithmic identification of optimal operating conditions, such as temperature and composition of solvent mixtures. It is relatively common to mix solvents to utilise the combined solvent properties,^56^–^60^ and some methods of determining composition of solvent blends exist.^61^ At this stage it is appropriate to treat the physical system as a ‘black box’ using concentrations of solvents as continuous variables.

We found that the combined properties of 1-octanol and triethyl amine, produced superior results to either of the pure solvents (see Table S4†), and that the combined solvent *σ* profile fits the predictive models discussed earlier, where the combined *σ* profile is a linear combination of the concentrations of the two individual ones, as shown in Fig. S9.† In addition to temperature and the two solvents mentioned, we included two further solvents – 1-nonanol and tributyl amine – as optimisation variables to investigate the temperature-dependent mixed solvents' landscape of amines and alcohols, with the aim of identifying promising recipes and temperature operating conditions. The trade-off between reactivity and selectivity makes the temperature landscape complex and non-intuitive. Algorithm modifications are described in Materials and methods.

**Fig. 5:**
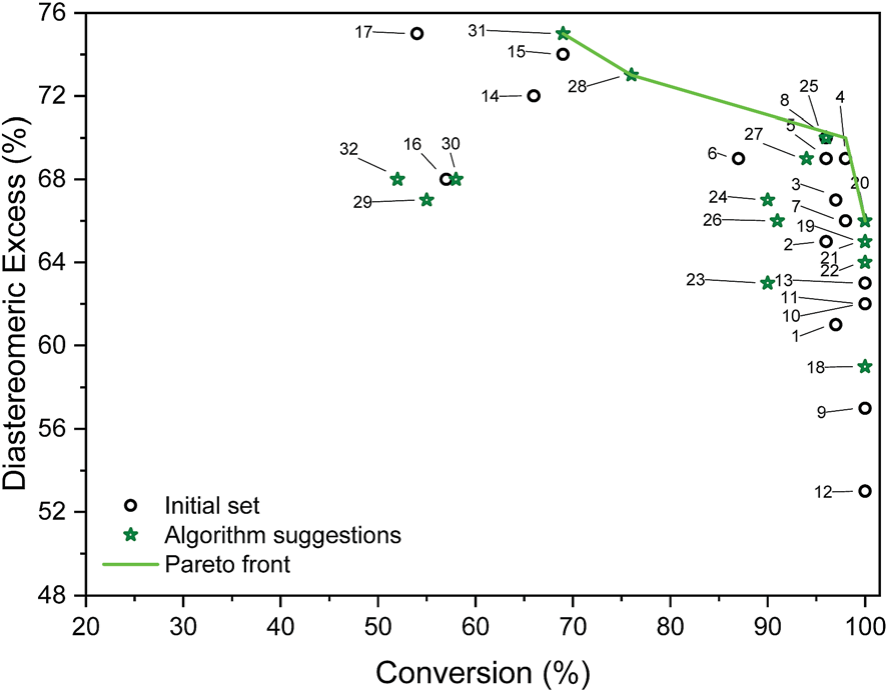
Outcomes of initial and algorithm-suggested solvent mixtures and the determined Pareto front. Labels refer to experiments (entries in Table S5†).

**Fig. 6:**
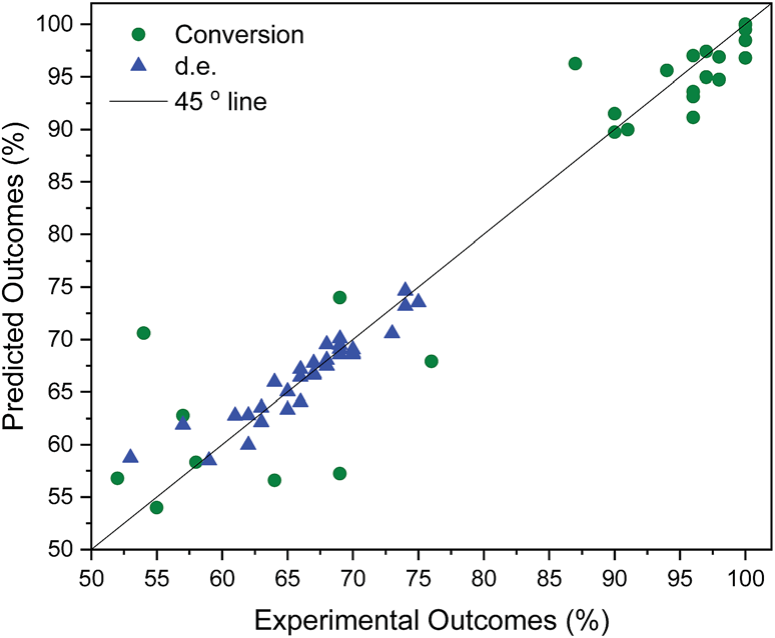
Cross-validation of solvent mixing results. *q*_conversion_^2^ = 0.91. *q*_d.e._^2^ = 0.88.

Table S4† shows the suggested recipes. The first five suggested reactions are at 82 °C, which was a temperature that achieved full conversion in all suggestions. In four out of five cases, an even higher selectivity than the training set entries that achieve full conversion. In a second algorithm-guided batch experiment, five mixtures at 65 °C were selected, showing in one case 96% and 70% (entry 25 in Table S4†). Three iterations were conducted, and outcomes of these recipes are shown in Fig. 5. The hyperparameters of the more accurate GP model (trained on all the available data) show that temperature is the most impactful variable towards each objective. Furthermore, alcohol content impacts conversion more than it does d.e., and that amine content impacts d.e. more. Out of the five recipes that lie on the determined Pareto front (entries 4, 20, 25, 28, 31 in Table S4†), four were selected by the algorithm. Cross-validated results show excellent GP predictive ability (*q*_conversion_^2^ = 0.91 and *q*_d.e._^2^ = 0.88, shown in Fig. 6). The advantage of this approach was the lack of significant *a priori* knowledge, whereas the descriptors approach described earlier gives detailed explanation of the importance of certain descriptors, giving further mechanistic insight into the reaction. Unlike in the case of other descriptors, it is straightforward to compute *σ*-profiles for solvent mixtures using a linear weighting of the pure solvents' *σ*-profiles. This is an advantage that allows predicting outcomes of solvent mixtures using models such as those discussed earlier in the paper.

### Automating machine learning pipelines using descriptors and classification

**Fig. 7:**
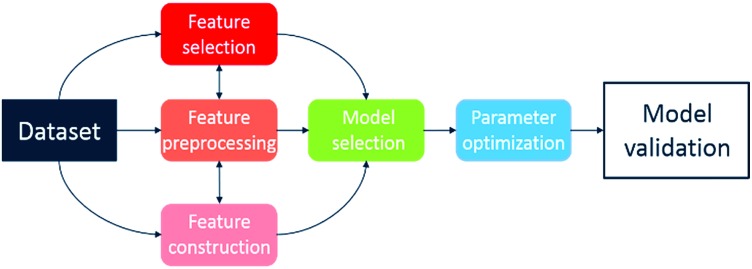
Illustration of machine learning pipeline workflow.

Recently, several automated machine learning strategies have been developed. Tree-based Pipeline Optimisation Tool (TPOT), a genetic programming-based method,^53^ is one such method and has proven to be a powerful tool for automating one of the most tedious parts of machine learning-pipeline design. A typical machine learning algorithm may be built with a pipeline as shown in Fig. 7. At each step, there are various possible choices to make, such as how to pre-process the data, what machine learning model to choose, and what hyperparameters to use.

**Fig. 8:**
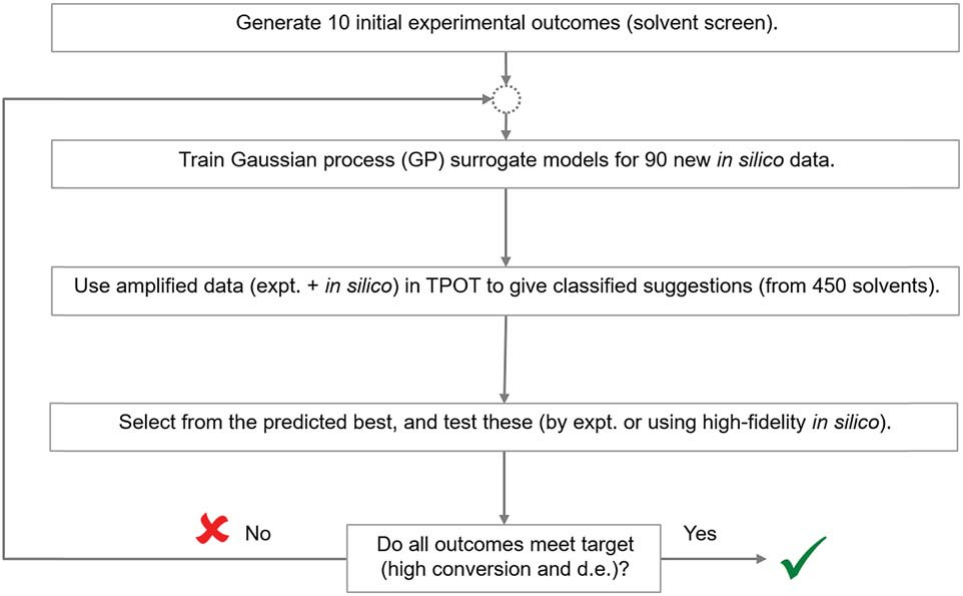
Strategy to investigate the applicability of Tree-based Pipeline Optimisation Tool (TPOT) to the solvent selection problem.

**Fig. 9:**
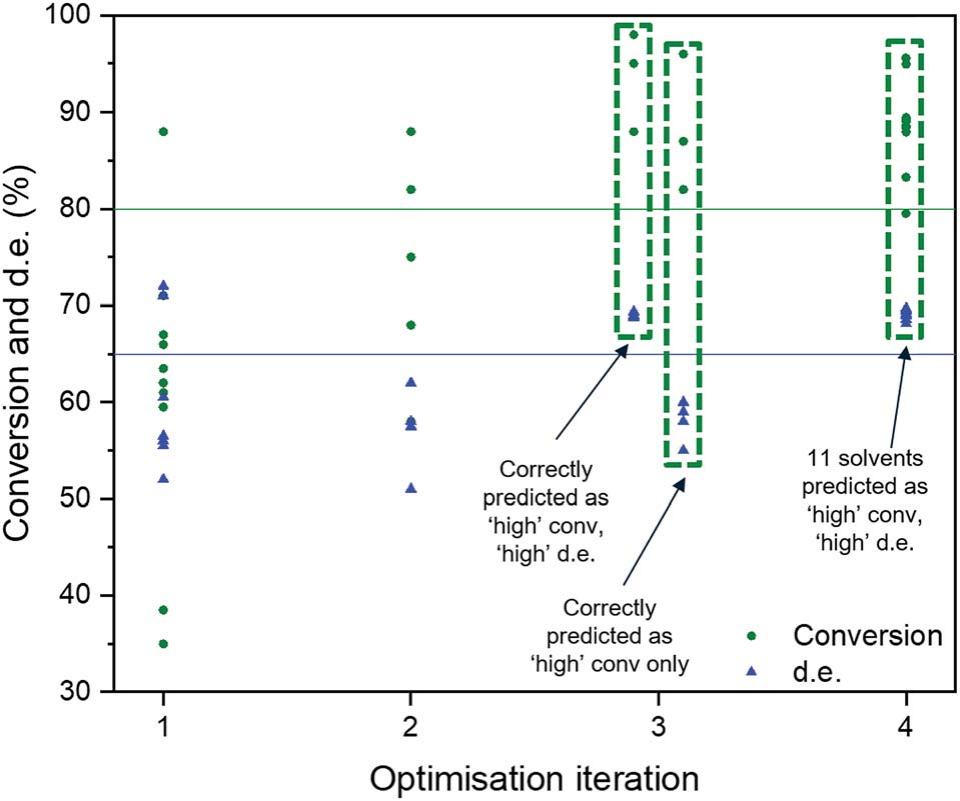
Results from the TPOT approach. Data labels correspond to the different solvents.

We adapted the pipeline optimization for our given problem domain. The aim was to determine its utility as a classification method, in conjunction with *in silico* modelling to amplify data, to navigate the descriptor space, and to optimise for superior solvents. The thresholds were set to 80% and 65% for conversion and d.e. respectively. The workflow is illustrated in Fig. 8. Given the large amount of data required to be used in classification and genetic programming, we decided to amplify a small set of 10 experimental data points to 100 by using the best GP surrogate models described earlier in the paper, specifically model 4 for conversion and model 1 for d.e. TPOT was then used to select some new solvents, which were tested experimentally and used to improve the accuracy of the amplified data by retraining the GP surrogate model. This loop is repeated until superior solvents are consistently found. The results show that this method rapidly classifies the experimentally confirmed excellent solvents as shown in Fig. 9, which shows reaction outcomes for the set of the initial 10 solvents in the first iteration (as individual data points). The experimentally verified solvents from Iteration 2 were used to improve the amplified data, which leads to very well-classified experimentally confirmed results in Iteration 3. Finally, these were incorporated into the feedback loop, and all solvents classified as ‘high’ were simulated in the highest fidelity versions of models 1 and 4 (for d.e. and conversion respectively), confirming excellent classification accuracy. Table S5,† which includes solvents described previously using TS-EMO, shows the detailed outcomes. Tables S6–S9† show the hyperparameters of models used in each iteration. Details of the best pipelines found, as well as classification accuracy at each iteration, are found in the ESI† as well.

## Conclusions

In conclusion, we have developed a new hybrid mechanistic-machine learning based method for rational solvent selection. This incorporates physically meaningful solvent descriptors with a Gaussian process-based algorithm, which has led to the rapid identification of promising solvents in asymmetric hydrogenation, outperforming those selected by human intuition in terms of conversion and diastereomeric excess. Screening charge density is shown to be an information-rich solvent descriptor for conversion and especially for diastereomeric excess. Using a dimensionality-reduced set of 17 physiochemical descriptors produces better models for conversion than those based only on screening charge density (cross-validation correlation coefficients of 0.84 and 0.76 respectively). Over 15 solvents with >90% conversion were algorithm-inspired, whereas only one such solvent existed in the training set suggested by a human expert. Using black-box optimisation we identified a set of optimal operating conditions and successfully explored the idea of using mixed solvents to attain the range of experimental space not accessible through pure solvents. Furthermore, the automated machine learning work-flow was successfully utilised for the problem of solvent selection. However, this approach is data-hungry and was supplemented with the statistically predictive surrogate model. In other circumstances, a predictive mechanistic model may be used instead of a statistical model for the same purpose. Ultimately, we expect the bridging between chemical information and data intensive machine learning methods to continue to advance. This opens the door for process chemists to adopt efficient robotic workflows in process development, saving time and resources, and freeing up researchers to make new discoveries.

## Conflicts of interest

There are no conflicts to declare.

## Supplementary Material

Supplementary information
