# Supplementary material for: Machine learning and molecular descriptors enable rational solvent selection in asymmetric catalysis
Source: Chem Sci. 2019 May 30;10(27):6697–706. doi: 10.1039/c9sc01844a (PMC6625492; doi:10.1039/c9sc01844a)
Supplement: Supplementary file 1 [file SC-010-C9SC01844A-s001.pdf]

Electronic Supplementary Material (ESI) for Chemical Science.
This journal is © The Royal Society of Chemistry 2019

CONFIDENTIAL

Machine learning and molecular descriptors enable rational solvent selection in asymmetric catalysis

Yehia Amar,[a] Artur M. Schweidtmann,[b] Liwei Cao,[a] Paul Deutsch,[c] and Alexei Lapkin[a,d*]

[a] *Department of Chemical Engineering and Biotechnology, University of Cambridge, Philippa Fawcett Drive, Cambridge, CB3 0AS, United Kingdom*

[b] *Aachener Verfahrenstechnik – Process Systems Engineering, RWTH Aachen University, Aachen, Germany*

[c] *UCB Pharma S.A. Allée de la Recherche, 60 1070 Brussels, Belgium*

[d] *Cambridge Centre for Advanced Research and Education in Singapore Ltd, 1 Create Way, CREATE Tower #05-05, 138602, Singapore*

Supporting Information

Contents:

1. Test set of Gaussian processes (GP) models for conversion and d.e.
2. New solvents identified, and experimental outcomes.
3. Cross validations using different models.
4. Experimental set-up illustration.
5. Solvent mixture recipes.
6. TPOT suggested recipes and outcomes.

In addition to this file, an Excel spreadsheet is available, with the library of solvents and their descriptors available.

* Corresponding author. Email: aal35@cam.ac.uk

1. Test set of GP models for conversion and d.e.

The developed surrogate model, trained on reaction outcomes of the 25 solvents shown in Figure 2, and using descriptors of model 1 (see Table 1), was used to predict outcomes on a test set of 9 separate solvents selected at random from the initial human-selected set, see Figure S1. Then a new surrogate model was trained on further data – the outcomes of the algorithm-identified experimentally tested solvents dibutyl amine, methyl octanoate, eucalyptol, and ethyl acetate – showing that the predictive performance has gone from poor (Figure S1, test set of algorithm trained on 25 initial data) to excellent (Figure S2, model trained on 29 data, 25 initial + first suggested 4) through retraining on the new data suggested.

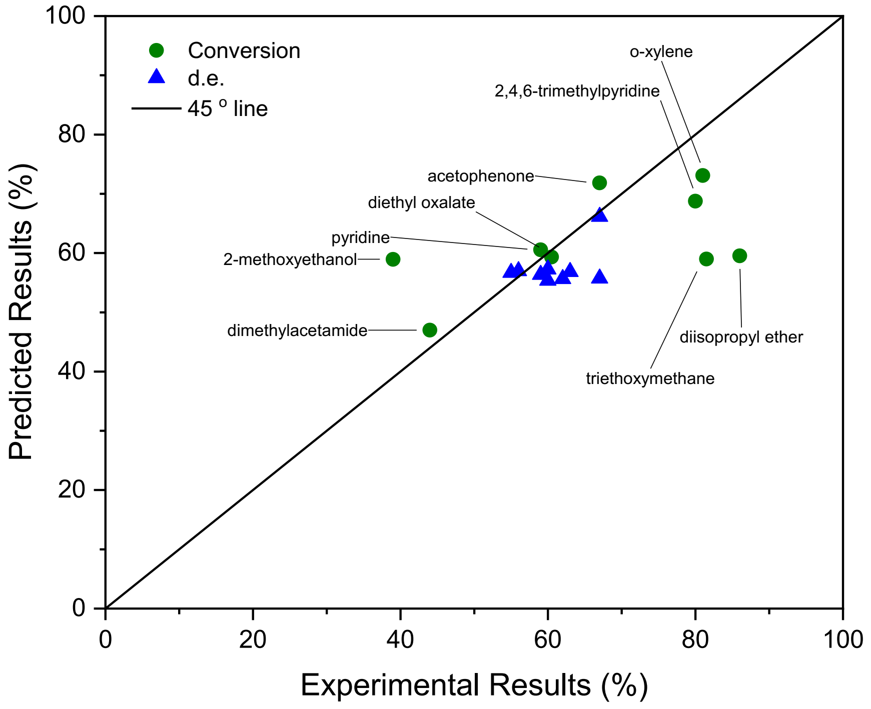

Figure S1. Results on test set using initial model using Model 1.

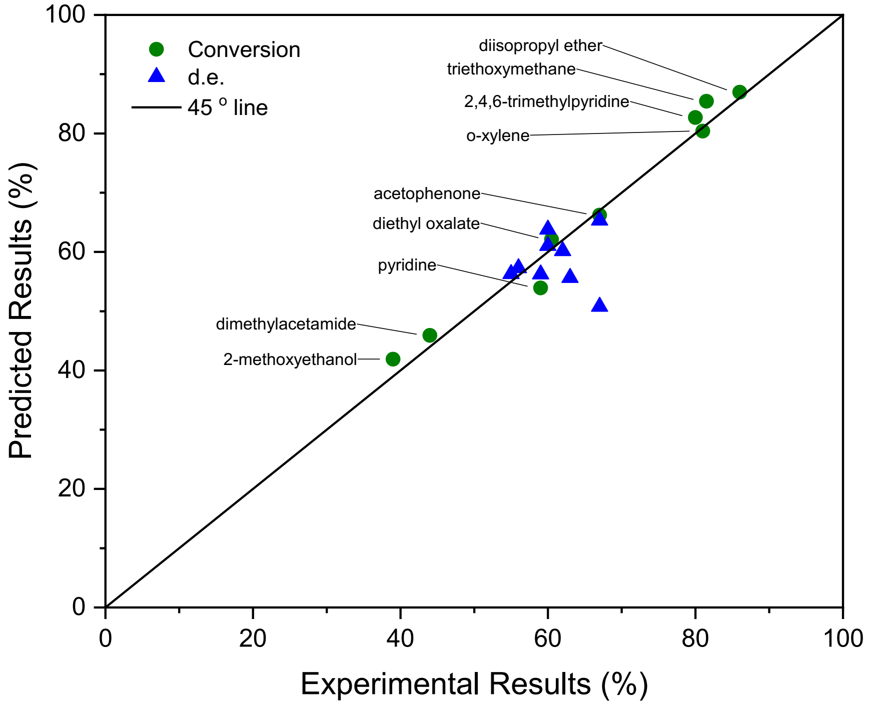

Figure S2. Model after first four suggested solvents are included in training (using Model 1).

Figure S3 shows the model predictions using model 2, trained on the initial 25 solvents, showing that the model is better than the initial model predictions using Model 1. Figure S4 shows model predictions using Model 4.

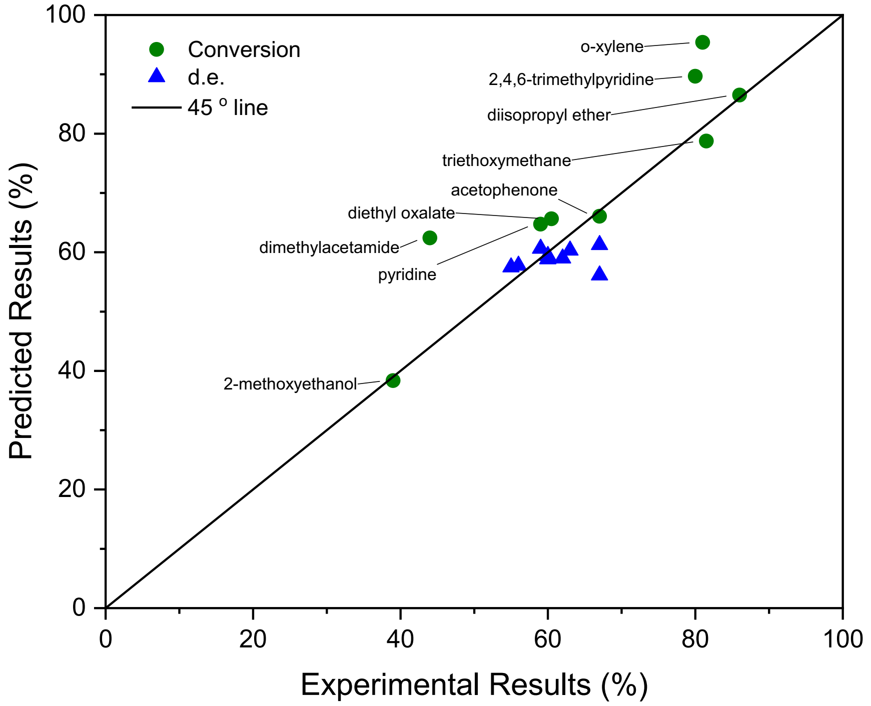

Figure S3. Initial model predictions (Model 2).

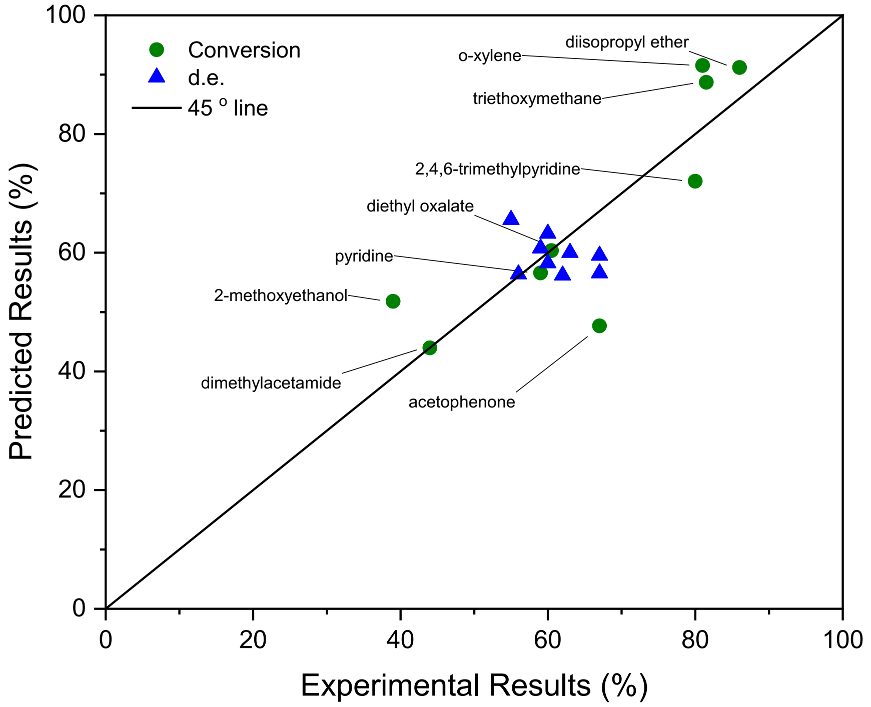

Figure S4. Initial model predictions (Model 3).

2. New solvents identified using TS-EMO, and outcomes

Table S1. New solvents selected using Model 1 DoE approach.

Entry	Solvent	Measured conversion (%)	Measured d.e. (%)
**1**	dibutyl amine	90	68
**2**	ethyl acetate	55	55
**3**	eucalyptol	96	56
**4**	methyl octanoate	92	58
**5**	aniline	69	67
**6**	methyl pentanoate	87	60
**7**	propyl propanoate	68	59
**8**	butyronitrile	68	51

Table S2. New solvents selected using Model 2 DoE approach.

Entry	Solvent	Measured conversion (%)	Measured d.e. (%)
**1**	methyl pentanoate	87	60
**2**	propyl propanoate	68	59
**3**	5-nonanone	90	56
**4**	1-nonanol	96	70
**5**	butyronitrile	68	51
**6**	tert-butylamine	75	62

Table S3. New solvents selected using Model 3 DoE approach.

Entry	Solvent	Measured conversion (%)	Measured d.e. (%)
**1**	propyl propanoate	68	59
**2**	2,6-dimethyl-4-heptanone	92	57
**3**	butyronitrile	68	51
**4**	2,4-dimethyl pentane	94	64
**5**	2,3-dimethyl pentane	93	60
**6**	propyl benzene	95	60
**7**	cumene	95	59
**8**	mesitylene	92	60
**9**	tributyl amine	98	66

3. Cross validations using different models

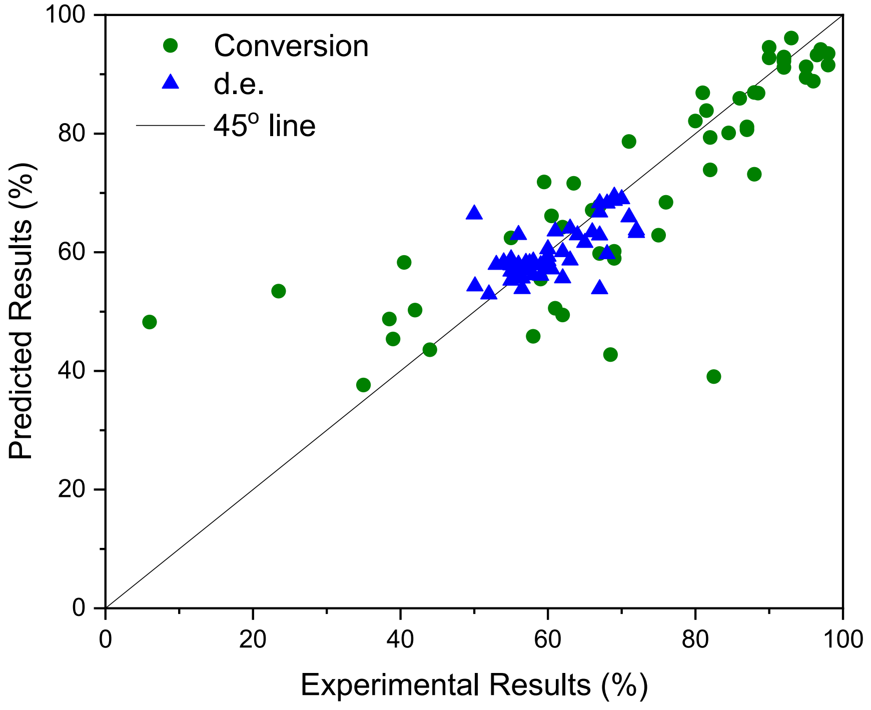

Figure S5. Leave-one-out cross-validation using Model 1.

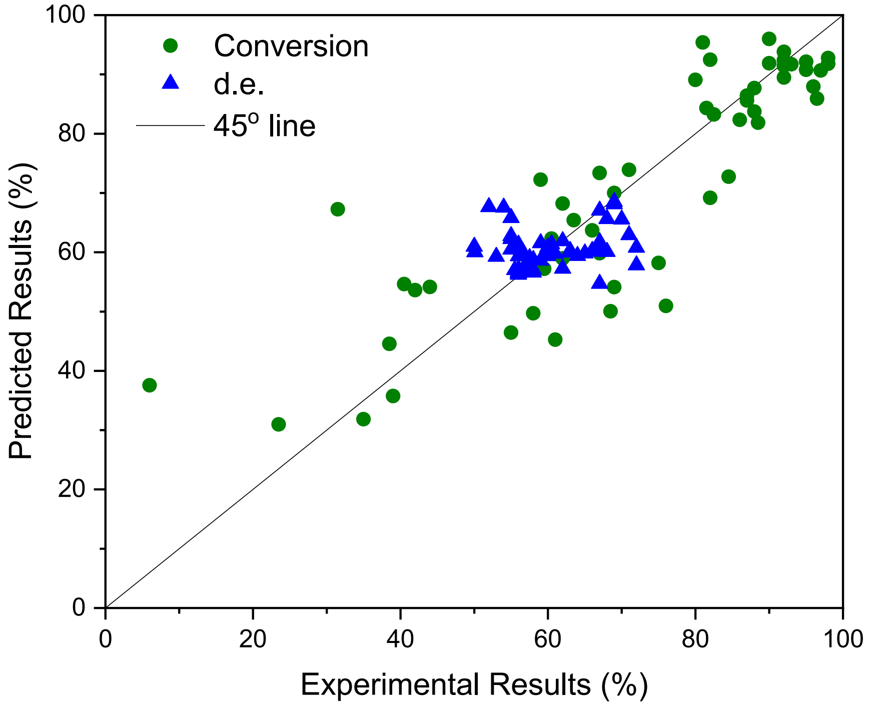

Figure S6. Leave-one-out cross-validation using Model 2.

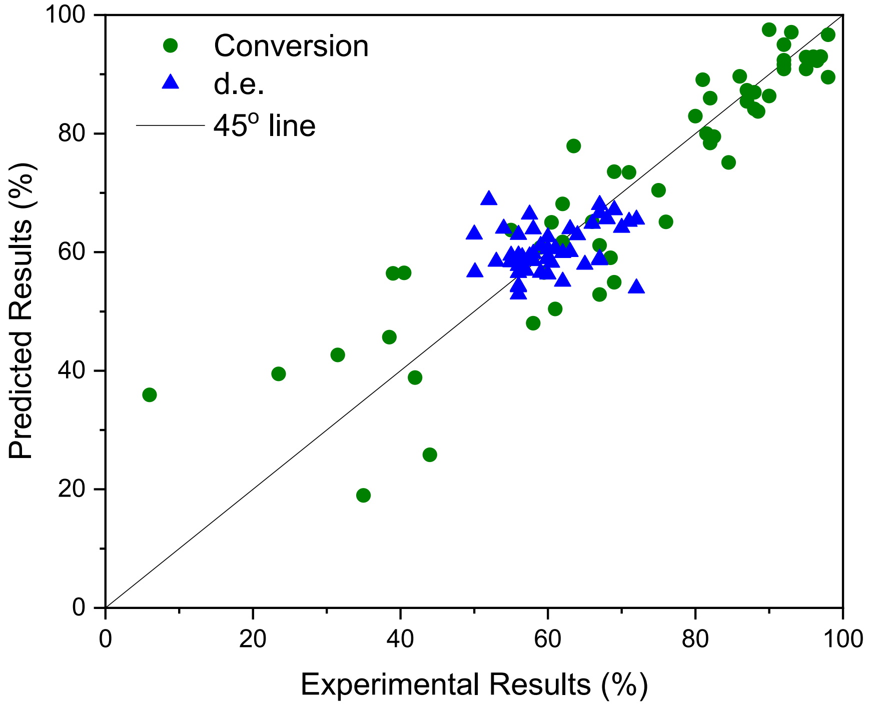

Figure S7. Leave-one-out cross-validation using Model 3.

4. Experimental set-up illustration.

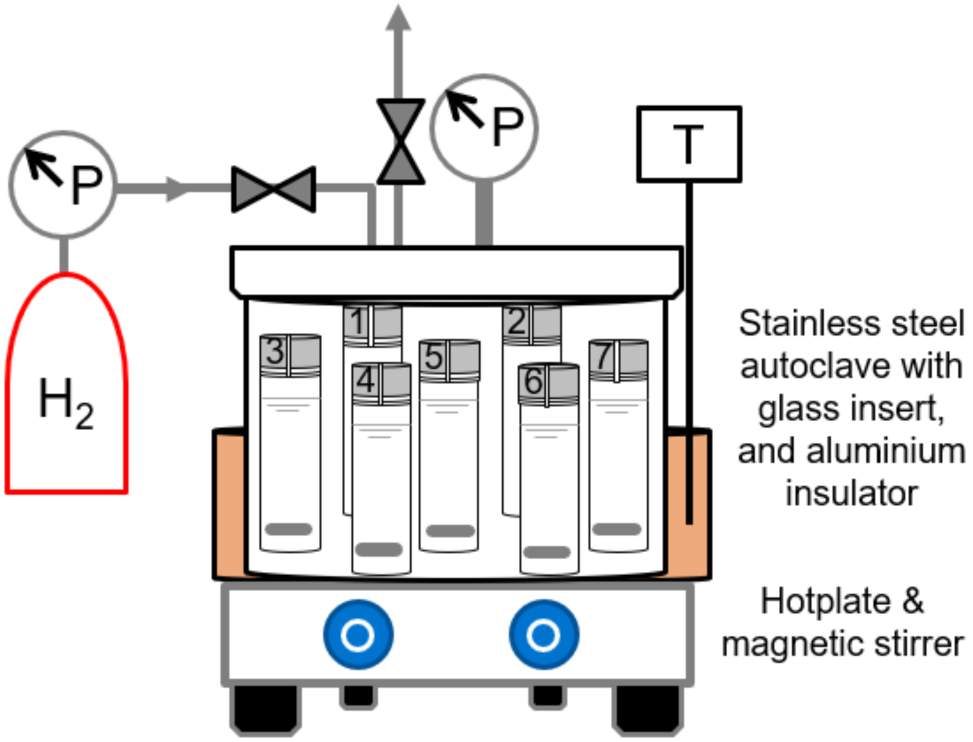

Figure S8. Illustration of experimental kit.

5. Solvent mixture recipes.

*Table S4. Solvents mixing recipes and outcomes. Pareto front in green. x_1 = triethyl amine, x_2 = 1-octanol, x_3 = tributyl amine, x_4 = 1-nonanol. * algorithm-determined.*

Entry	T (°C)	x_1	x_2	x_3	x_4	conv. (%)	d.e. (%)
1	70	1.00	-	-	-	97	61
2	70	0.80	0.20	-	-	96	65
3	70	0.50	0.50	-	-	97	67
4	70	0.20	0.80	-	-	98	69
5	70	0.10	0.90	-	-	96	69
6	70	-	1.00	-	-	87	69
7	70	-	-	1.00	-	98	66
8	70	-	-	-	1.00	96	70
9	90	-	-	1.00	-	>99	57
10	90	-	-	0.50	0.50	>99	62
11	90	-	-	-	1.00	>99	62
12	90	1.00	-	-	-	>99	53
13	90	-	1.00	-	-	>99	63
14	50	0.80	0.20	-	-	64	70
15	50	0.20	0.80	-	-	69	74
16	50	1.00	-	-	-	57	68
17	50	-	1.00	-	-	54	74
18*	82	0.81	0.13	0.06	-	>99	59
19*	82	-	0.65	0.25	0.10	>99	65
20*	82	-	0.58	0.30	0.12	>99	66
21*	82	0.01	0.66	0.19	0.14	>99	65
22*	82	-	0.56	0.30	0.14	>99	64
23*	65	0.99	-	0.01	-	90	63
24*	65	0.64	0.30	0.01	0.05	90	67
25*	65	0.21	0.56	0.22	0.01	96	70
26*	65	0.69	0.26	0.05	-	91	66
27*	65	0.17	0.50	0.24	0.09	94	69
28*	52	0.08	0.07	0.86	0.03	76	73
29*	52	0.93	0.01	0.05	0.01	55	67
30*	52	0.67	0.01	0.25	0.07	58	68
31*	52	-	0.12	0.07	0.81	69	75
32*	52	0.81	-	0.14	0.05	52	68

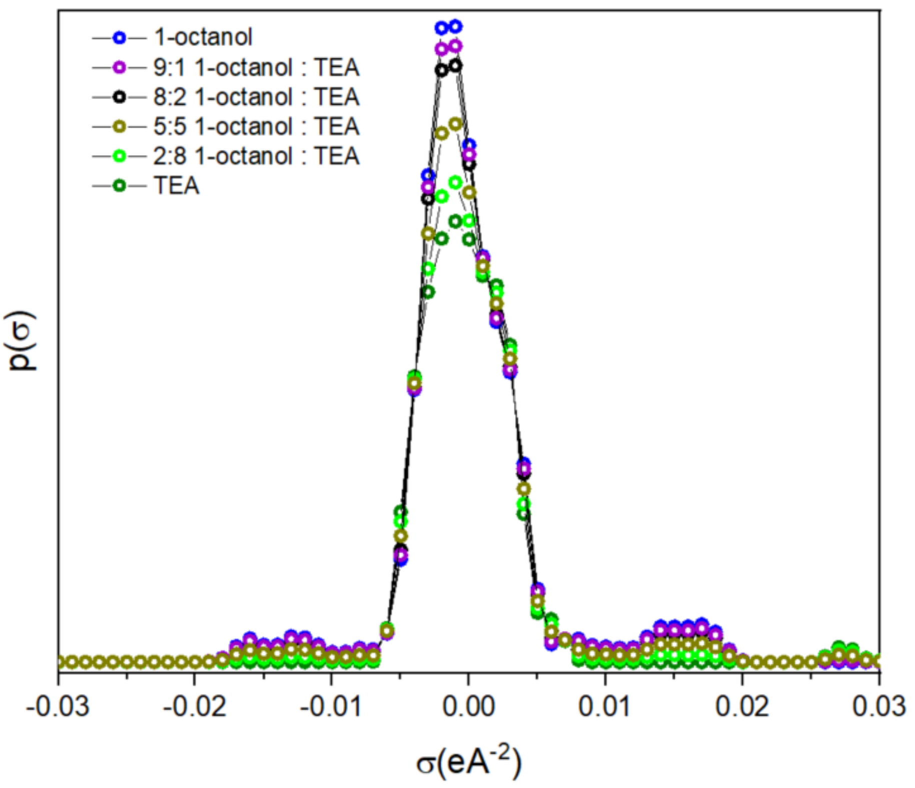

Figure S9. σ-profiles of the mixtures as a linear combination of pure component profiles.

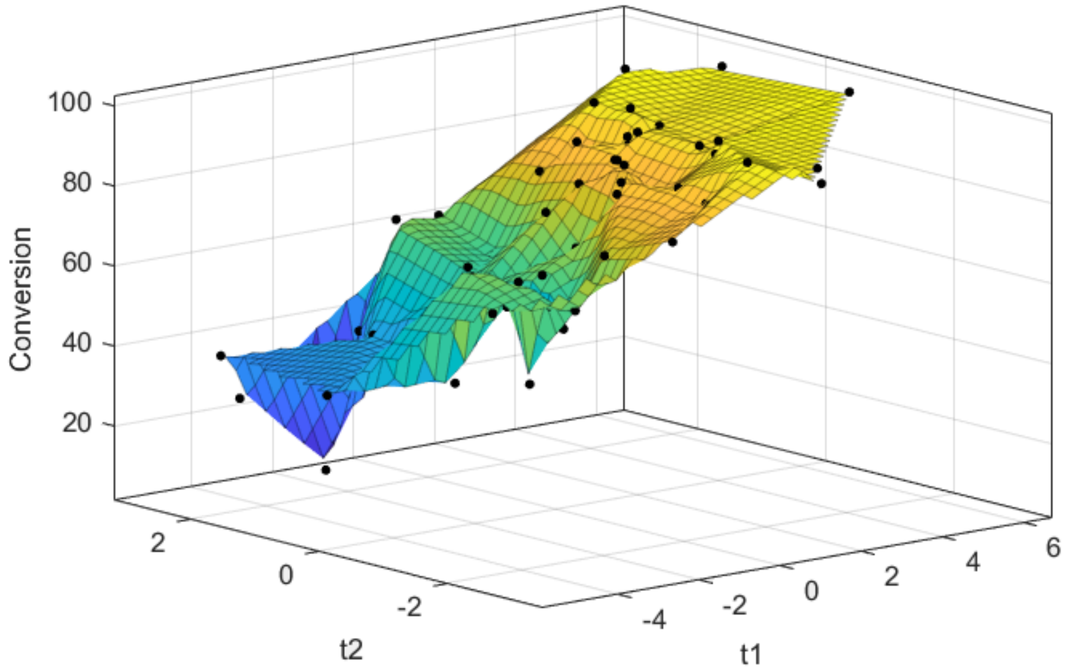

Figure S10. Visualisation of conversion (%) in different solvents (data points) vs. t_1 and t_2.

6. TPOT suggested recipes and outcomes

*Table S5. TPOT suggestion solvents method results. * simulated.*

Entry	Iteration	Solvent	Outcome (conv), %	Outcome (d.e.), %
1	1	2-butanol	61.0	71.0
2	1	2-pentanone	66.0	56.0
3	1	4-methyl-2-pentanone	71.0	56.0
4	1	2-propanone	62.0	56.0
5	1	cyclohexanone	59.5	55.5
6	1	2-butanone	67.0	56.0
7	1	toluene	88.0	56.5
8	1	acetonitrile	38.5	52.0
9	1	diethyl carbonate	63.5	60.5
10	1	dimethyl sulfoxide	35.0	72.0
11	2	diethyl ether	82.0	57.5
12	2	butyronitrile	68.0	51.0
13	2	octylamine	88.0	58.0
14	2	2-methyl-2-propanamine	75.0	62.0
15	2	dimethyl carbonate	58.0	58.0
16	3	1-nonanol	95.0	69.0
17	3	undecanol	98.0	69.0
18	3	octanol	88.0	69.0
19	3	4-methylanisole	96.0	60.0
20	3	1-methoxy-2-methylbenzene	87.0	59.0
21	3	N,N-dimethylaniline	87.0	58.0
22	3	methyl dodecanoate	82.0	55.0
23*	4	1-dodecanol	95.58	69.66
24*	4	2-methyl-1-heptanol	88.62	68.98
25*	4	2-methyl-3-heptanol	89.03	68.16
26*	4	2-octanol	87.96	69.73
27*	4	3-heptanol	83.28	69.16
28*	4	3-octanol	88.41	69.01
29*	4	4-heptanol	83.28	69.08
30*	4	4-octanol	89.12	68.97
31*	4	decanol	94.95	69.44
32*	4	heptanol	79.51	68.59
33*	4	4-methyl-3-heptanol	89.45	69.48

Table S6. TPOT iteration 1 hyperparameters of conversion and d.e. models after training on 10 experimental data for amplification (prediction of 90 new data points).

Descriptors model	Variable	GP1	GP2
Model 4	t_1 (σ_2', R, v_M, lnP)	2.09	-
Model 4	t_2 (T_B, T_M)	1.72	-
Model 4	t_3 (ρ, σ_3')	1.64	-
Model 1	σ_1	-	11.70
Model 1	σ_2	-	15.52
Model 1	σ_3	-	19.52
Model 1	σ_4	-	0.34
Model 1	σ_5	-	8.46

TPOT result:

- Best pipeline: GradientBoostingClassifier(input_matrix, learning_rate=0.1, max_depth=3, max_features=0.35, min_samples_leaf=9, min_samples_split=4, n_estimators=100, subsample=1.0).
- Classification accuracy based on 10-fold cross validation: 0.73

Table S7. TPOT iteration 2 hyperparameters of conversion and d.e. models after training on 15 experimental data for amplification (prediction of 90 new data points).

Descriptors model	Variable	GP1	GP2
Model 4	t_1 (σ_2', R, v_M, lnP)	1.53	-
Model 4	t_2 (T_B, T_M)	2.34	-
Model 4	t_3 (ρ, σ_3')	4.28	-
Model 1	σ_1	-	0.98
Model 1	σ_2	-	13.06
Model 1	σ_3	-	10.39
Model 1	σ_4	-	1.62
Model 1	σ_5	-	1.20

TPOT result:

- Best pipeline: LinearSVC(CombineDFs(PolynomialFeatures(RFE(input_matrix, criterion=entropy, max_features=0.35, n_estimators=100, step=0.75), degree=2, include_bias=False, interaction_only=False), ZeroCount(input_matrix)), C=5.0, dual=False, loss=squared_hinge, penalty=l1, tol=1e-05)
- Classification accuracy based on 10-fold cross validation: 0.81

Table S8. TPOT iteration 3 hyperparameters of conversion and d.e. models after training on 22 experimental data for amplification (prediction of 90 new data points).

Descriptors model	Variable	GP1	GP2
Model 4	t_1 (σ_2', R, v_M, lnP)	1.27	-
Model 4	t_2 (T_B, T_M)	3.35	-
Model 4	t_3 (ρ, σ_3')	6.68	-
Model 1	σ_1	-	0.8836
Model 1	σ_2	-	7.5226
Model 1	σ_3	-	5.4401
Model 1	σ_4	-	1.9244
Model 1	σ_5	-	0.6448

TPOT result:

- GradientBoostingClassifier(OneHotEncoder(input_matrix, minimum_fraction=0.1, sparse=False), learning_rate=0.1, max_depth=2, max_features=1.0, min_samples_leaf=19, min_samples_split=15, n_estimators=100, subsample=0.75)
- Classification accuracy based on 10-fold cross validation: 0.94

Table S9. Hyperparameters of conversion and d.e. models based on 58 data to create highest-fidelity models for simulation of all data points in iteration 4.

Descriptors model	Variable	GP1	GP2
Model 4	t_1 (σ_2', R, v_M, lnP)	1.33	-
Model 4	t_2 (T_B, T_M)	2.80	-
Model 4	t_3 (ρ, σ_3')	2.19	-
Model 1	σ_1	0.04	-
Model 1	σ_2	-	0.44
Model 1	σ_3	-	0.41
Model 1	σ_4	-	1.66
Model 1	σ_5	-	3.76
